# Programmed death receptor 1 (PD‐1) ligand Fc fusion proteins reduce T‐cell proliferation *in vitro* independently of PD‐1

**DOI:** 10.1111/imcb.12714

**Published:** 2023-12-09

**Authors:** Melissa Biemond, David Vremec, Daniel HD Gray, Philip D Hodgkin, Susanne Heinzel

**Affiliations:** ^1^ Immunology Division Walter and Eliza Hall Institute of Medical Research Parkville VIC Australia; ^2^ Department of Medical Biology The University of Melbourne Parkville VIC Australia; ^3^ Present address: Department of Immunology Leiden University Medical Center Leiden The Netherlands

**Keywords:** CD8^+^ T cells, immune checkpoints, naïve T cell activation, PD‐1

## Abstract

Programmed death receptor 1 (PD‐1) is an inhibitory receptor on T cells shown to restrain T‐cell proliferation. PD‐1 immune checkpoint blockade has emerged as a highly promising approach in cancer treatment. Much of our understanding of the function of PD‐1 is derived from *in vitro* T‐cell activation assays. Here we set out to further investigate how T cells integrate inhibitory signals such as PD‐1 *in vitro* using the PD‐1 agonist, PD‐1 ligand 1 (PD‐L1) fusion protein (PD‐L1.Fc), coimmobilized alongside anti‐CD3 agonist monoclonal antibody (mAb) on plates to deliver PD‐1 signals to wild‐type and PD‐1^−/−^ CD8^+^ T cells. Surprisingly, we found that the PD‐L1.Fc fusion protein inhibited T‐cell proliferation independently of PD‐1. This PD‐L1.Fc inhibition was observed in the presence and absence of CD28 and interleukin‐2 signaling. Binding of PD‐L1.Fc was restricted to PD‐1–expressing T cells and thus inhibition was not mediated by the interaction of PD‐L1.Fc with CD80 or other yet unknown binding partners. Furthermore, a similar PD‐1–independent reduction of T‐cell proliferation was observed with plate‐bound PD‐L2.Fc. Hence, our results suggest that the coimmobilization of PD‐1 ligand fusion proteins with anti‐CD3 mAb leads to a reduction of T‐cell engagement with plate‐bound anti‐CD3 mAb. This study demonstrates a nonspecific mechanism of T‐cell inhibition when PD‐L1.Fc or PD‐L2.Fc fusion proteins are delivered in a plate‐bound coimmobilization assay and highlights the importance of careful optimization of assay systems and reagents when interpreting their influence on T‐cell proliferation.

## Graphical abstract

In this work, we report a nonspecific, programmed death receptor 1 (PD‐1)–independent inhibition of T‐cell proliferation by PD‐L1.Fc (PD‐L1 coupled to the Fc portion of IgG1) and PD‐L2.Fc fusion proteins when coimmobilized with anti‐CD3 antibodies on tissue culture plates.

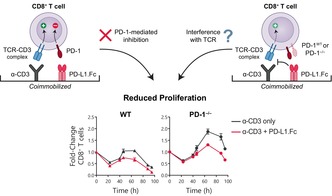

## INTRODUCTION

The clonal expansion of naïve T cells encountering cognate antigen underpins adaptive immune responses to infections and cancer. T cells rapidly expand to form a pool of antigen‐specific T cells that ideally mediate robust immunity against pathogens and tumors. A fine balance must be struck. If the T‐cell response is too weak, pathogens may persist and drive chronic inflammation. Too strong, and the response itself may cause life‐threatening immune pathology. Such precise modulation of the response magnitude is directed by the integration of a range of stimulatory and inhibitory signals. Indeed, inhibitory signals, such as those provided by the engagement of programmed death receptor 1 (PD‐1), have garnered much interest with the discovery that cancers can hijack the regulatory function of these receptors to avoid elimination by the immune system.^1^ Hence, targeting these inhibitory interactions has dramatically changed the approach to cancer treatment, yet the precise mechanisms by which engagement of inhibitory receptors restrain T‐cell responses remains unclear.

T‐cell responses to stimulation *in vitro* and *in vivo* conform to identifiable rules that can be monitored and described within a quantitative and predictive framework.^2^, ^3^, ^4^, ^5^ The size and duration of the division burst can ultimately be described by underlying independent proliferation parameters including entry into division, subsequent division rate, cell survival and the number of times the cells divide before returning to quiescence (termed division destiny).^2^, ^3^, ^4^, ^5^, ^6^ Using this framework, we have previously demonstrated that activating inputs increase the overall size of the T‐cell response *via* linear summation of the individual input effects on division destiny.^3^

Given our discovery of how costimulatory signals were integrated by T cells, we next wanted to explore how inhibitory receptor signaling is incorporated to determine the overall outcome of stimulation. We used PD‐1 as an example of a prototypical inhibitory signal due to its well‐established effect as an immune checkpoint.^1^, ^7^, ^8^, ^9^ Moreover, agonist and blocking reagents are readily available for the study of PD‐1. A fusion protein comprising the extracellular domain of PD‐1 ligand 1 (PD‐L1) coupled to the Fc portion of IgG1 (PD‐L1.Fc) is a PD‐1 agonist widely used *in vitro*.^9^, ^10^, ^11^, ^12^, ^13^, ^14^ It has been reported that PD‐L1.Fc binding to PD‐1 mimics the PD‐L1–PD‐1 binding interaction, inducing downstream signaling during T‐cell activation.^9^, ^10^ Early studies using this reagent indicated that signaling through PD‐1 may intersect with both the T‐cell receptor (TCR) complex and CD28 signaling pathways.^10^, ^15^ These and other signaling studies indicate that the proximity of PD‐1 to the TCR signaling complex and localization in the immune synapse are important for its ability to induce an inhibitory signal.^11^, ^16^, ^17^ Therefore, delivery of PD‐L1.Fc in the context of the TCR signal appears critical for inducing the inhibitory mechanism of PD‐1. As such, coimmobilization of anti‐CD3 monoclonal antibody (mAb) alongside PD‐L1.Fc on polystyrene tissue culture plates is commonly used to investigate PD‐1 signaling in T cells.^9^, ^18^, ^19^, ^20^, ^21^ This approach has the benefit of being easy to control, flexible in the amount of stimulus being delivered and able to be combined with other signaling inputs, apparently making it ideal for inclusion into existing quantitative *in vitro* T‐cell assay systems.

In this study, we aimed to incorporate PD‐1 signaling into our established *in vitro* T‐cell activation assays using PD‐1 ligand fusion proteins as a well‐characterized agonist. When coimmobilized with anti‐CD3 mAb, we found that PD‐L1.Fc reduced T‐cell proliferation; however, this effect was nonspecific and not mediated *via* the PD‐1 receptor. These findings highlight important caveats to the use of *in vitro* systems where PD‐L1.Fc is delivered with plate‐bound anti‐CD3 mAb. They indicate that careful interpretation is needed to distinguish genuine inhibitory signaling *via* the PD‐1 receptor *versus* artifacts arising from the assay system itself.

## RESULTS

### PD‐L1.Fc inhibits CD8^+^ T‐cell proliferation in a PD‐1–independent manner

To study the effect of PD‐1 signaling on mouse CD8^+^ T‐cell activation, 96‐well flat‐bottomed tissue culture plates were coated with anti‐mouse CD3 mAb (10 μg mL^−1^) alone or together with PD‐L1.Fc (10 μg mL^−1^; ACRObiosystems). Coimmobilization of these concentrations of anti‐CD3 mAb and PD‐L1.Fc was previously reported to have an inhibitory effect on T‐cell proliferation in a plate‐bound stimulation system.^9^ CellTrace Violet (CTV)–labeled wild type (WT) C57Bl/6 or C57Bl/6 *Pdcd1* ^*−/−*^ (PD‐1^−/−^) CD8^+^ T cells were stimulated on anti‐CD3 mAb‐coated plates with or without PD‐L1.Fc. Costimulation *via* CD28 was provided by the addition of soluble anti‐CD28 agonist mAb. Importantly, CD8^+^ T cells activated under these conditions produce interleukin‐2 (IL‐2), which induces potent autocrine survival and proliferation effects. Control of potential variation in endogenous IL‐2 levels and the downstream effects of IL‐2 in different culture conditions is crucial to disentangle the precise effects of individual signals.^22^ Hence, endogenously produced IL‐2 was neutralized by the addition of anti‐mouse IL‐2 mAb (clone S4B6).

**Figure 1:**
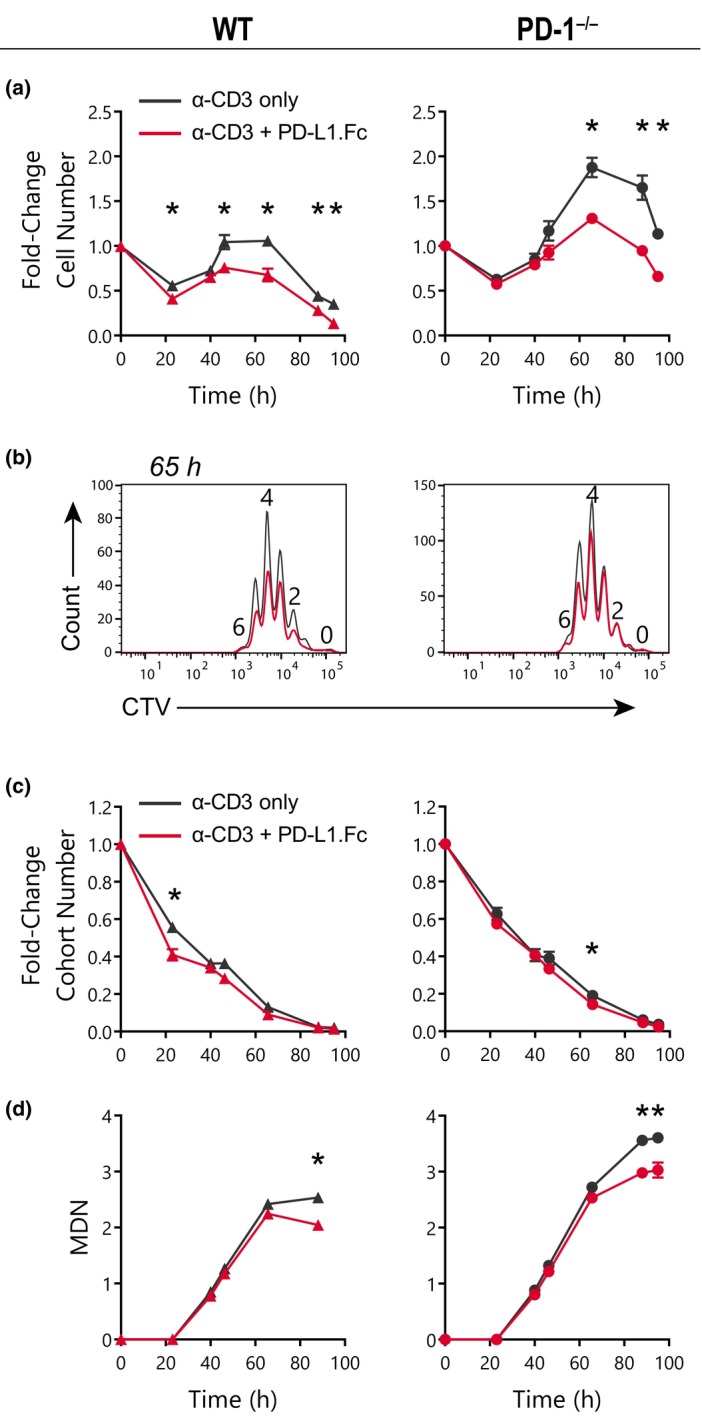
PD‐L1.Fc inhibits CD8^+^ T cells in a programmed death receptor 1 (PD‐1)–independent manner. CD8^+^ T cells were isolated from wild‐type (WT) and *Pdcd1* ^*−/−*^ (PD‐1^−/−^) mice and stimulated on plates coated with 10 μg mL^−1^ anti‐CD3 monoclonal antibody (mAb) ± 10 μg mL^−1^ PD‐L1.Fc. CD28 costimulation was provided by the addition of 6.23 μg mL^−1^ anti‐CD28 agonist mAb, clone 37.51. Endogenous interleukin (IL)‐2 was neutralized with 25 μg mL^−1^ anti‐IL‐2 mAb, clone S4B6. The data shown are representative of four independent experiments. **(a)** Cell number over time after stimulation with anti‐CD3 only (*black*) or anti‐CD3 + PD‐L1.Fc (*red*) (mean ± standard error of the mean, triplicate wells). **(b)** CellTrace Violet (CTV) division profile at 65 h after activation (one representative of triplicate wells). **(c, d)** Analysis of the total original cohort and mean division number (MDN) over time (mean ± standard error of the mean of three triplicate wells). Unpaired *t*‐tests were performed comparing conditions at each timepoint in **a, c** and **d**. *P < 0.05. PD‐L1, PD‐1 ligand 1; PD‐L1.Fc, PD‐L1 coupled to the Fc portion of IgG1.

As expected, PD‐L1.Fc coimmobilized with anti‐CD3 mAb‐reduced T‐cell expansion compared with T cells cultured with anti‐CD3 mAb alone (Figure 1a). Surprisingly, however, inhibition by PD‐L1.Fc was also observed in PD‐1^−/−^ T cells (Figure 1a). CTV histograms revealed that PD‐L1.Fc caused a mild reduction in proliferation (Figure 1b). This effect was further quantified using the precursor cohort analysis method, which adjusts the number of cells in each CTV division peak to separate the survival and division parameters of the founder cohort over time.^23^, ^24^ This analysis revealed that PD‐L1.Fc had a negligible impact on the survival of both WT and PD‐1^−/−^ CD8^+^ T cells (Figure 1c), and the overall inhibition in both genotypes was driven by a decrease in the mean division number (MDN) achieved before reaching a plateau that indicates a return to quiescence (i.e. division destiny; Figure 1d). These data indicate that the decrease in proliferation caused by PD‐L1.Fc influenced proliferation parameters in a similar manner in both WT and PD‐1^−/−^ T cells. We also confirmed that PD‐L1.Fc from a different provider (BioLegend) yielded the same results (data not shown). Of note, the overall expansion and MDN reached for PD‐1^−/−^ CD8^+^ T cells was greater than those observed in WT CD8^+^ T cells (Figure 1). This increased proliferative potential of PD‐1^−/−^ CD8^+^ T cells is consistent across experiments.

**Figure 2:**
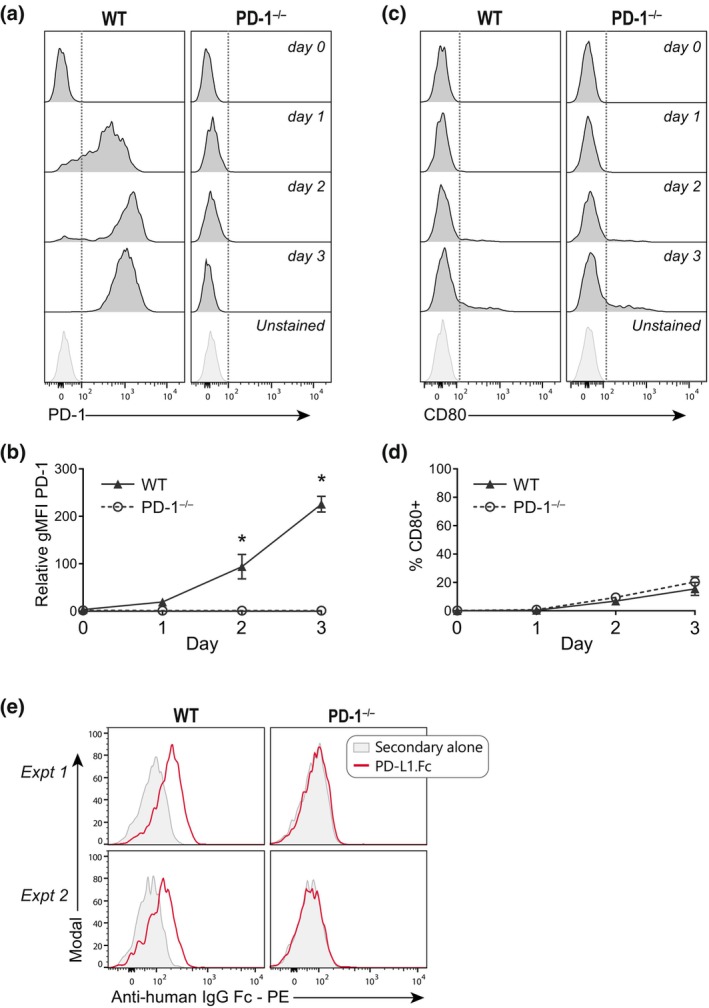
PD‐L1.Fc does not inhibit CD8^+^ T cells *via* specific surface receptor interactions. **(a–d)** Programmed death receptor 1 (PD‐1) and CD80 expression on wild‐type (WT) and PD‐1^−/−^ CD8^+^ T cells following anti‐CD3 + anti‐CD28 stimulation as in Figure 1. **(a)** Representative histograms of PD‐1 expression over time. **(b)** Summary of PD‐1 geometric mean fluorescence intensity relative to unstained in WT (*triangles*) and PD‐1^−/−^ (*open circles*) T cells in four independent experiments (mean ± standard error of the mean). **(c)** Representative histograms of CD80 expression over time. **(d)** Summary of % CD80^+^ T cells in WT (*triangles*) and PD‐1^−/−^ (*open circles*) T cells in three independent experiments (mean ± standard error of the mean). **(e)** Detection of PD‐L1.Fc binding to WT *versus* PD‐1^−/−^ CD8^+^ T cells day 2 after activation with anti‐CD3 + anti‐CD28 (two independent experiments). Unpaired *t*‐tests were performed in **b** and **d** comparing WT *versus* PD‐1^−/−^ T cells. *P < 0.05. Ig, immunoglobulin; PD‐L1, PD‐1 ligand 1; PD‐L1.Fc, PD‐L1 coupled to the Fc portion of IgG1; PE, phycoerythrin.

Given the unexpected inhibition of PD‐1^−/−^ T cells by PD‐L1.Fc, we investigated how this nonspecific inhibition may arise. First, we measured cell surface expression of PD‐1 to confirm PD‐1 deficiency in T cells from the PD‐1^−/−^ mice. Upregulation of PD‐1 was detected after activation of WT cells only, confirming the loss of PD‐1 in T cells from PD‐1^−/−^ mice (Figure 2a, b). We next considered whether the inhibition of proliferation in CD8^+^ T cells from both WT and PD‐1^−/−^ mice could be mediated by a specific interaction of PD‐L1.Fc with another receptor on T cells. A previous study demonstrated that PD‐L1.Fc delivered in the context of anti‐CD3 mAb on plates and on beads also inhibited PD‐1^−/−^ T cells.^13^ The authors went on to demonstrate that CD80 expressed on T cells was able to bind to PD‐L1.Fc to induce an inhibitory signal in T cells, and that this accounted for PD‐L1.Fc inhibition of PD‐1^−/−^ T cells.^13^ It is now known that CD80 chiefly interacts with PD‐L1 in *cis* on the cell surface.^25^, ^26^, ^27^ Nevertheless, PD‐L1.Fc may still have the ability to influence CD80 signaling *via* plate–cell interfaces. To explore this possibility, CD80 expression was measured on WT and PD‐1^−/−^ T cells before and after activation with anti‐CD3 and anti‐CD28 mAbs. We were unable to detect CD80 expression in naïve T cells and, once activated, few T cells upregulated CD80 (Figure 2c, d). Thus, PD‐L1–CD80 interactions are unlikely to explain the PD‐L1.Fc inhibition of PD‐1^−/−^ T cells we observed. We next considered the possibility that PD‐L1.Fc may interact with another, unidentified receptor on T cells to mediate T‐cell inhibition. To investigate this notion, we tested whether PD‐L1.Fc is capable of binding to PD‐1^−/−^ T cells. Using indirect immunofluorescence assayed by flow cytometry, we detected PD‐L1.Fc bound only to WT cells but not to PD‐1^−/−^ cells (Figure 2e), suggesting that PD‐L1.Fc does not bind another cell surface receptor on PD‐1^−/−^ cells. Collectively, these data indicate that PD‐L1.Fc does not inhibit WT and PD‐1^−/−^ T‐cell proliferation by a ligand–receptor‐specific mechanism.

**Figure 3:**
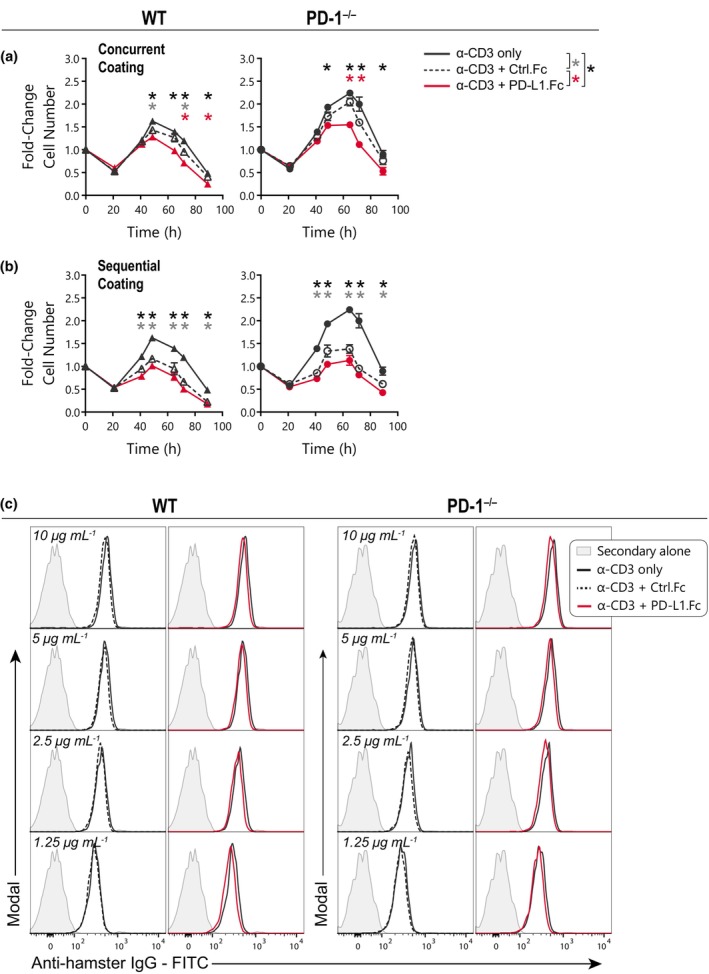
Protein concentration does not account for PD‐L1.Fc inhibition. **(a, b)** Wild‐type (WT) and PD‐1^−/−^ CD8^+^ T cells were stimulated on plates coated with anti‐CD3 only (*black*), anti‐CD3 + Ctrl.Fc (*black, dashed‐open*) or anti‐CD3 + PD‐L1.Fc (*red*), with anti‐CD28 costimulation and endogenous interleukin (IL)‐2 neutralized with anti‐IL‐2 monoclonal antibody (mAb). Analysis of cell number over time (mean ± standard error of the mean, of triplicate wells) of cells stimulated on plates coated concurrently with anti‐CD3 mAb and Fc (*a*, representative of two independent experiments), or coated overnight (4°C) with anti‐CD3 mAb followed by 4 h (37°C) with Fc (*b*, one experiment). **(c)** Detection of anti‐CD3 mAb (1.25–10 μg mL^−1^) binding on naïve WT or PD‐1^−/−^ CD8^+^ T cells with co‐incubation of anti‐CD3 alone or with equivalent concentrations of Ctrl.Fc or PD‐L1.Fc (1 experiment). Unpaired *t*‐tests were performed comparing conditions at each timepoint in **a** and **b**. *P < 0.05. PD‐1, programmed death receptor 1; PD‐L1, PD‐1 ligand 1; PD‐L1.Fc, PD‐L1 coupled to the Fc portion of IgG1.

We next considered whether delivery of PD‐L1.Fc alongside anti‐CD3 mAb on plates could be facilitating this nonspecific inhibition of WT and PD‐1^−/−^ T cells. In a previous study using artificial antigen‐presenting cell (APC) bead systems, simultaneous addition of a fusion protein such as PD‐L1.Fc with anti‐CD3 mAb was shown to competitively reduce the amount of anti‐CD3 mAb on the beads, consequently reducing proliferation indirectly by reducing TCR signaling.^28^ Hence, to address the possibility of protein competition in our plate system, we introduced a control Fc to maintain a consistent protein concentration during plate coating. WT and PD‐1^−/−^ CD8^+^ T cells were cultured on plates coated with 10 μg mL^−1^ anti‐CD3 mAb alone or in combination with 10 μg mL^−1^ human IgG1‐Fc (Ctrl.Fc) or PD‐L1.Fc. Addition of Ctrl.Fc to anti‐CD3 mAb induced a small decrease in the overall expansion of both WT and PD‐1^−/−^ T cells (Figure 3a), indicating that the presence of an additional protein may slightly reduce the level of accessible anti‐CD3 mAb on the plate. However, the addition of Ctrl.Fc did not fully recapitulate the inhibition observed in the presence of PD‐L1.Fc in either WT or PD‐1^−/−^ T cells (Figure 3a). The authors of the aforementioned study went on to show that correction of anti‐CD3 mAb levels using a sequential coating protocol mitigated the reduced proliferation.^28^ However, when we adopted an analogous sequential plate coating protocol where plates were coated first with anti‐CD3 mAb before the addition of PD‐L1.Fc or Ctrl.Fc, we found that both Ctrl.Fc and PD‐L1.Fc inhibited both WT and PD‐1^−/−^ T‐cell proliferation to an even greater extent (Figure 3b). This suggests that while the amount of anti‐CD3 mAb bound to the plate may be varied by different co‐coating protocols, this does not account for the nonspecific inhibition of PD‐L1.Fc we detected using the standard protocol.

To test whether PD‐L1.Fc was able to directly reduce the binding of anti‐CD3 mAb to T cells we performed an indirect immunofluorescence assay. We did not detect any change in anti‐CD3 mAb binding to naïve WT and PD‐1^−/−^ CD8^+^ T cells incubated with soluble anti‐CD3 mAb alone, or with anti‐CD3 that was preincubated with soluble Ctrl.Fc or PD‐L1.Fc (Figure 3c), indicating that PD‐L1.Fc does not inherently interfere with anti‐CD3 mAb binding to T cells. Taken together, these results at first suggest that PD‐L1.Fc inhibition of WT and PD‐1^−/−^ T cells does not arise from altered total protein concentration during plate coating or by directly inhibiting anti‐CD3 binding to T cells.

### PD‐L1.Fc inhibits T‐cell proliferation in PD‐1^−/−^ T cells independent of IL‐2 production or CD28 signaling

**Figure 4:**
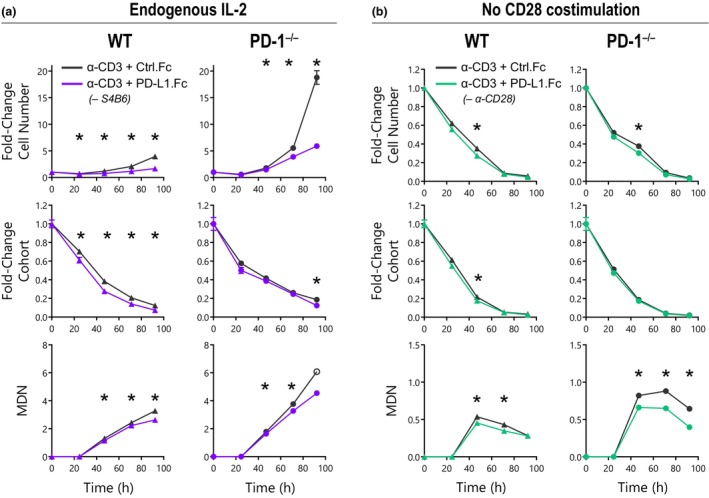
PD‐L1.Fc inhibits CD8^+^ T‐cell expansion irrespective of interleukin (IL)‐2 or CD28 costimulation. Wild‐type (WT) and PD‐1^−/−^ CD8^+^ T cells were stimulated on plates coated with anti‐CD3 + Ctrl.Fc or PD‐L1.Fc. Analysis of cell number, original cohort and mean division number (MDN) over time with Ctrl.Fc (*black*) or PD‐L1.Fc (*purple or green*) is shown. **(a)** Cell response in the absence of the anti–IL‐2 neutralizing monoclonal antibody (mAb) S4B6. Mean ± standard error of the mean of three triplicate wells, one experiment. The open symbol indicates an estimated MDN where MDN could not be accurately determined because of loss of CellTrace Violet (CTV) resolution. **(b)** Cell response in the absence of anti‐CD28 mAb 37.51 costimulation. Mean ± standard error of the mean of three triplicate wells, unpaired *t*‐tests were performed comparing conditions at each timepoint, *P < 0.05, representative of four independent experiments. PD‐1, programmed death receptor 1; PD‐L1, PD‐1 ligand 1; PD‐L1.Fc, PD‐L1 coupled to the Fc portion of IgG1.

Having ruled out several potential mechanisms thus far, we next considered whether the nonspecific PD‐L1.Fc inhibition of WT and PD‐1^−/−^ T‐cell proliferation could be a consequence of our activation conditions. The most fundamental difference between our experimental design and others reported previously^9^, ^10^, ^13^, ^14^ is the addition of anti–IL‐2 mAb to neutralize endogenously produced IL‐2. Autocrine IL‐2 production and signaling induce T‐cell proliferation and a feed‐forward loop.^29^ This effect can greatly magnify early impacts on T‐cell activation. Hence, neutralizing IL‐2 enables us to isolate the direct effect of specific signals on the T‐cell proliferation response without the amplifying effect of endogenous IL‐2 production. However, studies of PD‐1 function have suggested that the major mechanism of PD‐1–mediated inhibition of T cells is *via* the reduction of IL‐2 production.^10^, ^30^ Hence, neutralizing IL‐2 may be masking the PD‐1–dependent inhibitory effect of PD‐L1.Fc. To address this possibility, we cultured CD8^+^ T cells with PD‐L1.Fc without neutralizing IL‐2. As expected, both WT and PD‐1^−/−^ T cells proliferated strongly when IL‐2 was not neutralized (Figure 4a). As has been consistently observed in this study, PD‐1^−/−^ T cells expanded more than WT T cells (Figure 4a). This effect appears to have been amplified in the presence of endogenously produced IL‐2, again highlighting the importance of controlling these secondary effects by neutralizing IL‐2. Crucially, however, proliferation of PD‐1^−/−^ cells was still inhibited by PD‐L1.Fc (Figure 4a). Therefore, even in the presence of endogenously produced IL‐2, inhibition of T‐cell proliferation by PD‐L1.Fc was unrelated to PD‐1 expression.

Recent studies suggest that PD‐1 directly targets the CD28 pathway^17^ and therefore CD28 signaling would be required to resolve PD‐1–mediated inhibition. However, earlier studies reported that provision of CD28 costimulation overcame the inhibitory effect of PD‐L1.Fc,^9^ indicating that CD28 costimulation may again mask the PD‐1–dependent inhibitory effect of PD‐L1.Fc. Hence, to investigate whether PD‐1–specific inhibition with PD‐L1.Fc would be revealed in the absence of CD28 costimulation, WT and PD‐1^−/−^ T cells were stimulated without the addition of anti‐CD28 mAb. The removal of CD28 costimulation markedly attenuated the proliferative response overall, again with a greater expansion observed in PD‐1^−/−^ T cells (Figure 4b). PD‐L1.Fc only had a marginal effect on WT T cells, however, reduced proliferation was still observed in PD‐1–deficient T cells when PD‐L1.Fc was provided (Figure 4b). Thus, removal or inclusion of CD28 costimulation does not reveal PD‐1–specific inhibition of T cells by plate‐bound PD‐L1.Fc.

### Inhibition of PD‐1^−/−^ T‐cell proliferation by PD‐L2.Fc

**Figure 5:**
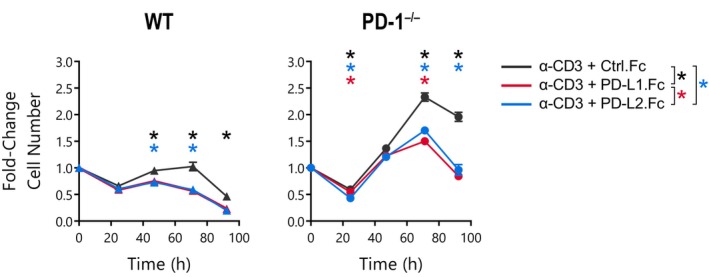
Equivalent, nonspecific inhibition of CD8^+^ T‐cell expansion by PD‐L1.Fc and PD‐L2.Fc fusion proteins. Number of wild‐type (WT) and PD‐1^−/−^ CD8^+^ T cells over time following activation with anti‐CD3 coimmobilized with Ctrl.Fc (*black*), PD‐L1.Fc (*red*) or PD‐L2.Fc (*blue*) in the presence of anti‐CD28 and anti‐IL‐2. Mean ± standard error of the mean of three triplicate wells, unpaired *t*‐tests were performed comparing conditions at each timepoint, *P < 0.05, one experiment. IL, interleukin; PD‐1, programmed death receptor 1; PD‐L1, PD‐1 ligand 1; PD‐L1.Fc, PD‐L1 coupled to the Fc portion of IgG1.

We further assessed whether a PD‐1–specific inhibitory signal could be detected using PD‐L2.Fc as an alternative ligand for PD‐1. The fusion protein, PD‐L2.Fc, has been demonstrated to bind to PD‐1 with an increased affinity compared with PD‐L1.Fc and to induce inhibition in T cells *via* PD‐1.^13^, ^31^, ^32^ CD8^+^ T cells from WT and PD‐1^−/−^ mice were stimulated on plates coated with 10 μg mL^−1^ anti‐CD3 mAb alone or in combination with 10 μg mL^−1^ of either Ctrl.Fc, PD‐L1.Fc or PD‐L2.Fc. We observed that PD‐L2.Fc induced a reduction in both WT and PD‐1^−/−^ T‐cell proliferation (Figure 5). Strikingly, PD‐L2.Fc and PD‐L1.Fc reduced the proliferation of WT and PD‐1^−/−^ CD8^+^ T cells to an almost identical level (Figure 5) despite the previously reported distinction in PD‐L1 and PD‐L2 function.^31^, ^32^

### The inhibitory effect of PD‐L1.Fc and PD‐L2.Fc is consistent across WT and PD‐1^−/−^ CD8^+^ T cells

**Figure 6:**
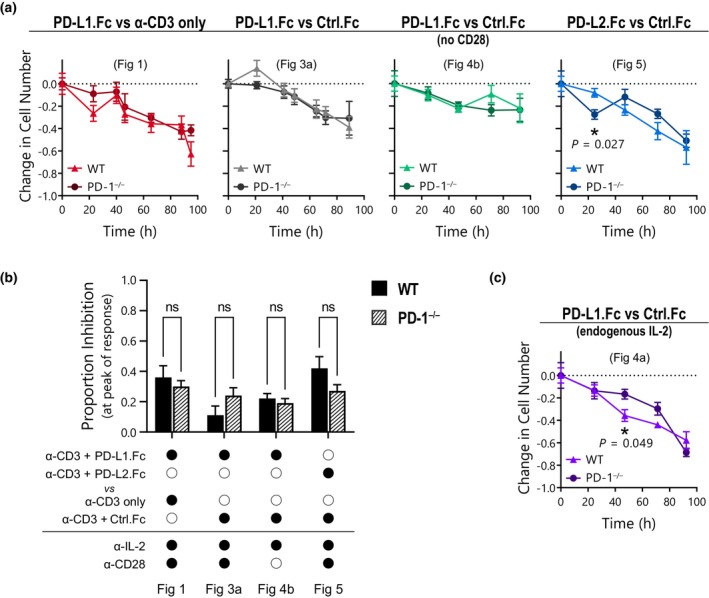
Programmed death receptor 1 (PD‐1) ligand fusion proteins inhibit wild‐type (WT) and PD‐1^−/−^ CD8^+^ T cells equivalently. Inhibition of overall cell number in Fc‐treated cultures from Figures 1 and 3, 4, 5 relative to anti‐CD3 only or anti‐CD3 + Ctrl.Fc controls. **(a, c)** Fold‐change in total cell number over time in WT (*triangles*) *versus* PD‐1^−/−^ (*circles*) CD8^+^ T cells. Mean ± standard error of the mean of three triplicate wells, multiple unpaired *t*‐tests performed comparing conditions at each timepoint, *P* > 0.05 except where indicated. **(b)** Proportion inhibition by PD‐1 ligand fusion proteins at the peak of the response in WT (*solid*) compared with PD‐1^−/−^ (*striped*) T cells. Mean ± standard error of the mean of three triplicate wells, unpaired *t*‐tests were performed, ns = *P* > 0.05. PD‐L1, PD‐1 ligand 1; PD‐L1.Fc, PD‐L1 coupled to the Fc portion of IgG1.

Throughout this study, we have observed that PD‐L1.Fc induced consistent inhibition of WT and PD‐1^−/−^ CD8^+^ T‐cell proliferation under a variety of conditions tested. To further evaluate the reduction in proliferation across experimental conditions in Figures 1 and 3, 4, 5, the inhibitory effect was calculated as the fold‐change in overall cell number in cells treated with the PD‐1 ligand fusion protein compared with those in anti‐CD3 mAb alone or anti‐CD3 mAb with Ctrl.Fc control conditions. Analysis of the overall response over time reveals a remarkably similar pattern of inhibition between WT and PD‐1^−/−^ T cells in conditions where endogenous IL‐2 was controlled using the anti‐IL‐2 neutralizing mAb (Figure 6a). Further representation of the proportion inhibition of the overall cell number at peak expansion revealed no significant difference between WT and PD‐1^−/−^ T cells (Figure 6b, Table 1). As discussed earlier, the removal of the anti–IL‐2 neutralizing mAb amplified differences between WT and PD‐1^−/−^ T‐cell expansion kinetics in Figure 4a, and the peak of the response was not observed in the experiment timeframe. Thus, direct comparisons between genotypes were more difficult to interpret. Nonetheless, the fold‐change over time between anti‐CD3 mAb with Ctrl.Fc *versus* anti‐CD3 mAb with PD‐L1.Fc was still somewhat consistent between WT and PD‐1^−/−^ T cells despite the substantial variation in response magnitude (Figure 6c). These results strongly implicate a general, PD‐1–independent mechanism for the inhibition of T‐cell proliferation when PD‐L fusion proteins are delivered with anti‐CD3 mAb on tissue culture plates. Overall, these observations provide no evidence of an inhibitory effect of PD‐L fusion proteins that is mediated *via* the PD‐1 receptor.

**Table 1:** Proportion inhibition by PD‐1 ligand fusion proteins at the peak of the response.

	α‐CD3 + PD‐L1.Fc	α‐CD3 + PD‐L2.Fc	*Versus* α‐CD3 only	*Versus* α‐CD3 + Ctrl.Fc	With α‐IL‐2	With α‐CD28	Genotype	Peak TP (h)	Total cell number (mean)		Proportion inhibition	Difference	*P*
									Control	PD‐L.Fc	Mean ± standard error of the mean		
Figure 1	●	○	●	○	●	●	WT	65.6	7164	4605	0.36 ± 0.08	0.06	0.525
							PD‐1^−/−^	65.6	15 632	10 869	0.30 ± 0.04		
Figure 3	●	○	○	●	●	●	WT	48.7	10 281	9198	0.11 ± 0.06	−0.13	0.180
							PD‐1^−/−^	64.0	15 460	11 683	0.24 ± 0.05		
Figure 4a	●	○	○	●	○	●		*n/a*					
								*n/a*					
Figure 4b	●	○	○	●	●	○	WT	47.0	2137	1661	0.22 ± 0.03	0.03	0.544
							PD‐1^−/−^	47.0	3264	2627	0.19 ± 0.03		
Figure 5	○	●	○	●	●	●	WT	71.2	6239	3600	0.42 ± 0.08	0.15	0.154
							PD‐1^−/−^	71.2	20 235	14 794	0.27 ± 0.04		

Unpaired *t*‐tests were performed on the proportion inhibition by Fc fusion protein in WT compared with PD‐1^−/−^ T‐cell responses.

IL, interleukin; PD‐1, programmed death receptor 1; PD‐L1, PD‐1 ligand 1; PD‐L1.Fc, PD‐L1 coupled to the Fc portion of IgG1; TP, time point; WT, wild type.

## DISCUSSION

T cells are exposed to several different signals upon activation, which together determine the size and shape of the subsequent immune response. Dissecting the contribution of individual signals is essential to understanding how combinations of inputs are integrated. Hence, the ability to deliver individual stimuli in a controlled manner is crucial for these investigations. In this study, we used the PD‐1 agonists PD‐L1.Fc and PD‐L2.Fc to induce PD‐1 signaling in T cells. However, we found that, when coimmobilized with anti‐CD3 mAb on tissue culture plates, inhibition of CD8^+^ T cells by these reagents was independent of their target, PD‐1. This was a surprising finding given that PD‐1 ligand and, indeed, many other receptor–ligand fusion proteins are a widely used and published method for delivering signals to T cells *in vitro*.^9^, ^14^, ^18^, ^19^, ^20^, ^32^, ^33^

Many previous studies using PD‐1 ligand fusion proteins in plate‐based systems assessed the reagents’ inhibitory effects using methods such as ^3^H‐thymidine incorporation as a measure of proliferation or cytokine production at a single timepoint.^9^, ^14^, ^32^ These approaches may lack the sensitivity and temporal context to resolve nuanced quantitative changes in proliferation caused by altered signals. In the foundational study demonstrating the utility of PD‐L1.Fc as an agonist to induce PD‐1 signaling, PD‐L1.Fc was found to specifically inhibit WT and not PD‐1^−/−^ T‐cell proliferation as measured by ^3^H‐thymidine incorporation.^9^ Perhaps the most compelling point of difference for this study is that the assay was performed in whole T cells with no neutralization of endogenous IL‐2. Given that PD‐1 signaling affects IL‐2 production,^10^, ^30^ in coculture systems IL‐2 production by CD4^+^ T cells may interplay with the sensitivity of CD8^+^ T cells to IL‐2 to amplify any PD‐1–specific activity of PD‐L1.Fc. Interestingly, a subsequent study by the same group^13^ revealed that isolated CD4^+^ T cells from PD‐1–deficient mice did have reduced proliferation when treated with PD‐L1.Fc delivered in the context of anti‐CD3 mAb on plates and on artificial APC beads. This study suggested that a specific interaction of PD‐L1.Fc with CD80 expressed on activated T cells was responsible for the inhibitory effect of PD‐L1.Fc on PD‐1^−/−^ T cells.^13^ However, over the course of our investigations, we ruled out the expression of CD80 as a potential mediator for the nonspecific effect of PD‐L1.Fc. Furthermore, the same inhibition was observed when PD‐L2.Fc was used which, in contrast to PD‐L1, does not bind to CD80.^13^, ^26^ We also confirmed that PD‐L1.Fc only binds to WT but not PD‐1^−/−^ cells, ruling out a specific binding interaction with a yet unknown receptor for PD‐L1.

Given the consistency of the inhibitory effect observed under all conditions, we hypothesized that the inhibition of T cells might be a result of a reduction of TCR signaling. We found that plate‐bound PD‐L1.Fc restrained T‐cell expansion by primarily reducing the number of rounds of division the cells underwent. TCR and costimulatory signals such as CD28 or IL‐2 regulate the same proliferation parameters to influence T‐cell proliferative and cytokine responses,^3^, ^22^, ^23^, ^34^ making it difficult to disentangle the functional outcomes of interfering with any of those signals. Thus, reduced anti‐CD3 signaling may readily appear to be a reduction in costimulation or an increase in inhibitory signaling.

Consistent with a TCR‐mediated effect, a study using artificial APC beads reported that covalent coupling of anti‐CD3 mAb simultaneously with inhibitory ligand Fc fusion proteins (including PD‐L1.Fc) led to steric competition between the anti‐CD3 mAb and the fusion proteins.^28^ One possibility for our plate‐based system was that PD‐1 ligand fusion proteins similarly compete with anti‐CD3 mAb for binding sites during plate coating, reducing the amount of anti‐CD3 mAb bound to the plate. Interestingly, maintaining a consistent total protein concentration during plate coating using Ctrl.Fc as a control for PD‐L1.Fc did not fully recapitulate PD‐1 ligand Fc‐induced inhibition (Figure 3). Despite having the same formulation, the control and PD‐1 ligand fusion proteins used in our analysis have different molecular properties (e.g. different molecular mass). Thus, it is possible that the PD‐1 ligand fusion proteins are more effective competitors for plate‐binding sites than Ctrl.Fc. Alternatively, PD‐1 ligand Fc fusion proteins may have molecular properties that allow them to sterically interfere with TCR access to plate‐bound anti‐CD3 mAb. Although we demonstrated that PD‐L1.Fc did not directly interfere with anti‐CD3 mAb binding to TCR in a soluble format, this does not preclude this as a possibility at the plate surface. Interestingly, we found that immobilizing fusion proteins on plates after anti‐CD3 mAb coating did not reduce the nonspecific inhibitory effect of PD‐L1.Fc, but appeared to strengthen the inhibitory effect of Ctrl.Fc as well as PD‐L1.Fc. We speculated that coating anti‐CD3 mAb alone would eliminate competition for binding sites and ensure a consistent amount of anti‐CD3 mAb on the plate in each condition. Putative mechanisms for the increased inhibition when Ig fusion proteins were coated after anti‐CD3 mAb could be displacement of already bound anti‐CD3 by the fusion proteins, or augmentation of the ostensible steric interference by the Fc fusion proteins. Thus, our hypothesis that perturbation of the anti‐CD3 mAb interaction with TCR is inducing inhibition of T cells remains a strong possibility.

Consistent with our proposed mechanism, a recent study showed equal inhibition of interferon‐γ production in WT and PD‐1^−/−^ mouse T cells by PD‐L1.Fc when coimmobilized directly on tissue culture plates with anti‐CD3 mAb.^21^ Of note, the authors also showed that the delivery of soluble PD‐L1.Fc crosslinked with anti‐Fc did not deliver a PD‐1–specific signal,^21^ in line with evidence that anti‐CD3 and PD‐L1.Fc need to be coimmobilized together.^9^, ^11^, ^16^ Only optimization of anti‐CD3 mAb and PD‐L1.Fc concentrations and the introduction of a biotin–streptavidin binding interface on the plate surface to capture both reagents were able to alleviate the nonspecific effect.^21^

There are many different formulations of PD‐L1.Fc and Ctrl.Fc available and the formulations used in different studies vary and are sometimes not specified.^9^, ^18^, ^19^, ^20^, ^33^ Although we obtained the same results when using fusion proteins from a different supplier (data not shown), it cannot be excluded that differences in the manufacturing and formulation of the fusion proteins may contribute to different outcomes, potentially because of different steric properties. Nonetheless, our study strongly supports the need for careful optimization and adequate controls in assessing all plate‐based systems.

Taken together, our study underlines the difficulties in assessing the consequences of PD‐1 signaling using *in vitro* culture systems. Furthermore, it raises the question of where one can have confidence in *in vitro* T‐cell inhibition studies using these or similar reagents. Fortunately, the inhibitory function of PD‐1 has been extensively demonstrated *via* a number of other methods. For example, delivery of PD‐L1 with TCR signals on engineered cellular APCs induces inhibition of T‐cell proliferation and cytokine production *in vitro*.^10^, ^31^ Phenotypic studies of PD‐1–deficient mice reveal susceptibility to immune pathologies in the absence of PD‐1.^35^, ^36^, ^37^, ^38^ Artificial expression of PD‐L1 or administration of PD‐L1.Fc *in vivo* has shown protective effects against T‐cell–mediated pathology in models of type 1 diabetes, colitis, liver sepsis and nephritis.^14^, ^39^, ^40^, ^41^ Certainly, PD‐1 blockade has been widely demonstrated to increase T‐cell activity and enhance viral and tumor clearance, strongly indicating the blockade of an inhibitory signal.^7^, ^8^, ^42^ Thus, the role of PD‐1 as an inhibitory signal to T cells is undisputed. However, despite this consensus and the therapeutic potential of PD‐1 therapies, the exact mechanisms and consequences of PD‐1 signaling in specific T‐cell subsets remain to be fully elucidated. We have previously demonstrated that investigation of how various signal inputs quantitatively influence T‐cell proliferative outputs requires the resolution of expansion parameters in a controlled and reproducible *in vitro* cellular assay. This reductionist approach has played a pivotal role in building our quantitative understanding of T‐cell behavior *in vitro* and importantly allows accurate prediction of T‐cell behavior *in vivo*.^3^ However, our present study demonstrating a nonspecific, PD‐1–independent effect of PD‐L1‐Fc highlights significant obstacles to the use of *in vitro* approaches for studies investigating the function of PD‐1 signaling. These problems can be mitigated if careful attention is paid to the stimulation system, and critically PD‐1^−/−^ cells must be used as control to ensure allocated quantitative effects are attributable to the PD‐1 signal. Published studies without this control should be interpreted cautiously. Moreover, the development of a precise, rigorous and specific reductionist *in vitro* system will be paramount to the further dissection of the impact of PD‐1 inhibitory signaling in T‐cell expansion.

## METHODS

### Mice

C57Bl/6 mice were obtained from the Walter and Eliza Hall (WEHI) animal facility (Kew, Victoria, Australia). C57Bl/6 *Pdcd1* ^*−/−*^ (PD‐1–deficient) mice^43^ were obtained from Professor Arlene Sharpe (Harvard Medical School, Boston, MA, USA). Mice were housed under specific pathogen‐free conditions in the WEHI animal facility (Parkville, Victoria, Australia) and were used between 6 and 10 weeks of age. All experiments were approved by the WEHI Animal Ethics Committee.

### Fusion proteins

Murine PD‐L1 (19–238) coupled to human IgG1‐Fc (100–330, PD‐L1.Fc), human IgG1‐Fc (99–330, Ctrl.Fc) and murine PD‐L2 (20–219) coupled to human‐IgG1‐Fc (100–330) were obtained from ACRObiosystems (Newark, DE, USA). All fusion proteins were received lyophilized from a solution of 50 mM Tris and 100 mM glycine with 10% trehalose (pH 7.5) and reconstituted with sterile deionized water according to the manufacturer's instructions.

### Plate coating

CD8^+^ T cells were stimulated in 96‐well flat‐bottomed plates with immobilized anti‐mouse CD3 mAb (clone 145‐2C11; WEHI Antibody Facility, Parkville, Victoria, Australia). Flat‐bottomed plates were coated with 50 μL of 10 μg mL^−1^ anti‐CD3 mAb in Dulbecco's phosphate‐buffered saline (dPBS; Gibco, Waltham, MA, USA) either with or without 10 μg mL^−1^ Fc fusion protein (ACRObiosystems) as indicated. Plates were incubated overnight at 4°C, then washed twice with 200 μL dPBS (Gibco) and blocked by washing once with culture medium before the addition of T cells. For sequential coating, plates were first coated with 50 μL of 10 μg mL^−1^ anti‐CD3 mAb in dPBS (Gibco) and incubated overnight at 4°C. The plates were then washed two times with 200 μL dPBS (Gibco) before the addition of 10 μg mL^−1^ Fc fusion protein (ACRObiosystems). Plates were incubated for 4 h at 37°C, then washed twice with 200 μL dPBS (Gibco) and blocked by washing once with culture medium before the addition of T cells.

### Murine T‐cell culture

Inguinal, brachial, axillary and cervical lymph nodes (LNs) were dissected, pooled and single‐cell suspensions were made. CD8^+^ T cells were enriched using the EasySep pan‐CD8 negative isolation kit (STEMCELL Technologies, Vancouver, Canada) according to the manufacturer's instructions. CD8^+^ T cells were labeled with CTV proliferation tracking dye (Invitrogen, Carlsbad, CA, USA) at 5 μM in PBS supplemented with 0.1% bovine serum albumin (Sigma, St Louis, MI, USA) for 20 min at 37°C.

CD8^+^ T cells were seeded at a density of 1 × 10^4^ cells per well in 200 μL RPMI (Roswell Park Memorial Institute) 1640 medium (Gibco) supplemented with 10% fetal calf serum (Sigma), non‐essential amino acids (Invitrogen), 1 mM sodium pyruvate (Invitrogen), 10 mM HEPES (4‐(2‐hydroxyethyl)‐1‐piperazineethanesulfonic acid; Invitrogen), 2 mM GlutaMAX (Invitrogen), 100 U mL^−1^ penicillin and 100 mg mL^−1^ streptomycin (Invitrogen) and 50 mM β‐mercaptoethanol (Sigma). Culture media was further supplemented with anti‐mouse CD28 mAb (clone 37.51; WEHI Antibody Facility) and 25 μg mL^−1^ neutralizing anti‐mouse IL‐2 mAb (clone S4B6; WEHI Antibody Facility), except where indicated. Cell cultures were counted once or twice daily, a minimum of 8 h apart. For quantification of live cells, 10^4^ rainbow calibration beads (BD Biosciences, Franklin Lakes, NJ, USA) and 0.2 μM propidium iodide (Sigma) were added to each well before data acquisition on the BD FACS Canto II or FACS Fortessa X20 system (Becton Dickinson, Franklin Lakes, NJ, USA). Gating and analysis were performed using FlowJo version 10 (Becton Dickinson).

### Cell surface staining

PD‐1 expression was detected using anti‐mouse PD‐1 APC (clone 29F.1A12; BioLegend, San Diego, CA, USA) and CD80 was detected using anti‐mouse CD80 PE (clone 16‐10.A1; BD Biosciences). Cells were processed and stained in fluorescence‐activated cell sorting (FACS) buffer comprising PBS supplemented with 0.1% bovine serum albumin (Sigma) and 0.1% sodium azide (Sigma). Cells were stained between 25 and 45 min on ice in the dark. Stained cells were analyzed on the FACS Fortessa X20 (Becton Dickinson).

### Indirect immunofluorescence assays

Binding of PD‐L1.Fc to activated CD8^+^ T cells was performed by harvesting cells from *in vitro* cultures and transferring them to a V‐bottomed plate. Cells were recovered by centrifugation (500 *g*, 5 min), then resuspended in Fixable Viability Dye eFluor 780 (1/1000; eBioscience, San Diego, CA, USA) in PBS and incubated for 10 min on ice. Cells were washed by the addition of FACS buffer and subjected to centrifugation (500 *g*, 5 min) before being resuspended in supernatant from the anti‐mouse CD16/CD32 hybridoma cell line 2′4G2 (1/10) in FACS buffer for 20 min to block Fc receptors. Cells were then centrifuged (500*g*, 5 min), resuspended in PD‐L1.Fc (100 μg mL^−1^; ACRObiosystems) and incubated for 45 min on ice in the dark. After incubation, cells were washed twice before being resuspended in phycoerythrin (PE) conjugated anti‐human IgG‐Fc (1/50; eBioscience). Cells were incubated in secondary antibody for 30 min on ice in the dark. Stained cells were then washed once with 180 μL of FACS buffer and centrifuged at 500*g* for 5 min and resuspended in 200 μL FACS buffer for acquisition on the FACS Canto II system (Becton Dickinson).

Detection of anti‐CD3 mAb binding was performed on naïve cells CD8^+^ T cells. Naïve WT and PD‐1^−/−^ CD8^+^ T cells were isolated and resuspended in Fixable Viability Dye eFluor 780 (1/1000; eBioscience) in PBS. After a 20‐min incubation on ice, cells were washed by the addition of FACS buffer and subjected to centrifugation (500*g*, 5 min). Hamster anti‐mouse CD3 (145‐2C11; WEHI Antibody Facility) was preincubated for 15 min on ice either alone or with Ctrl.Fc or PD‐L1.Fc (ACRObiosystems) at equal concentrations of anti‐CD3 and fusion protein ranging from 1.25 to 10 μg mL^−1^ each in FACS buffer. After washing, cells were incubated with the preincubated antibody mixes for 30 min on ice and then washed twice before being resuspended in anti‐hamster IgG FITC (1/100; BD Biosciences) to detect anti‐CD3 bound to the T cells. Cells were incubated for 30 min on ice in the dark, then washed once with 180 μL FACS buffer, centrifuged at 500*g* for 5 min and then resuspended in 200 μL FACS buffer for acquisition on the FACS Canto II (Becton Dickinson).

### Cell division and cohort analysis

Analysis of cell division was performed using flow cytometric analysis of CTV using FlowJo version 10 (Becton Dickinson). Live T cells were identified by gating on forward scatter and side scatter characteristics, followed by doublet exclusion based on forward scatter area *versus* forward scatter height, and exclusion of dead cells by propidium iodide staining. The number of rainbow beads acquired was used to determine the proportion of each well sampled. The total cell number per well was then calculated by multiplying the number of cells counted by the inverse of the proportion of beads counted:

$$\text{Total cell number}=\frac{\#\text{beads added}}{\text{beads counted}}\times \text{live cells counted}$$

The number of cells in each division was determined by gating division peaks in the histograms of log‐fluorescence of CTV, and again multiplying the cells in each division by the proportion of beads counted.

The total cell number was further interrogated using adaptations of the precursor cohort method as described previously.^23^, ^24^ The adaptation of the cohort analysis utilized here to visualize survival takes advantage of the discovery that division and death time are inherited in families and show clonal concordance.^44^, ^45^ The precursor cohort number is an estimate of number of original founder cells that are represented within a given generation (*i*) and was calculated as follows:

$$\begin{array}{l} \text{Cohort number}\;\mathrm{per}\;\text{generation,}C_{i}\\ \;=\frac{\text{number of cells in generation,}i}{2^{i}} \end{array}$$

The population cohort number is the sum of *C*_*i*_ from all resolvable generations (*I*):

$$\text{Cohort number}=\sum_{i=0}^{I}C_{i}$$

Following the cohort number over time indicates the inherited survival features of the population and can be visualized and fitted to a survival curve, typically conforming to a lognormal distribution.^4^, ^5^ The *C*_*i*_ can also be used to calculate the MDN.^3^ The MDN is the average number of divisions in the original cell cohort at each timepoint and was calculated as follows:

$$\mathrm{MDN}=\frac{\sum_{i=0}^{I}i\cdot C_{i}}{\sum_{i=0}^{I}C_{i}}$$

Plotting the MDN over time can allow visualization of proliferation parameters including time to first division, division rate, and number of divisions reached before cells return to quiescence (i.e. division destiny and the time at which this occurs).

### Statistical analysis

Unpaired parametric *t*‐tests were performed where indicated using GraphPad Prism version 9.5 (GraphPad Software, Boston, MA, USA). Conditions were compared at each individual timepoint, with no correction for multiple comparisons.

## AUTHOR CONTRIBUTIONS

**Melissa Biemond:** Conceptualization; formal analysis; investigation; methodology; writing – original draft; writing – review and editing. **David Vremec:** Methodology; writing – review and editing. **Daniel HD Gray:** Conceptualization; supervision; writing – review and editing. **Philip D Hodgkin:** Conceptualization; funding acquisition; methodology; supervision; writing – review and editing. **Susanne Heinzel:** Conceptualization; funding acquisition; supervision; writing – review and editing.

## CONFLICT OF INTEREST

The authors declare no conflict of interest.

## Data Availability

Data are available on request from the authors.
